# Supplementary material for: The AP-2 complex has a specialized clathrin-independent role in apical endocytosis and polar growth in fungi
Source: eLife. 2017 Feb 21;6:e20083. doi: 10.7554/eLife.20083 (PMC5338921; doi:10.7554/eLife.20083)
Supplement: Supplementary file 1. — DOI: http://dx.doi.org/10.7554/eLife.20083.022 [file elife-20083-supp1.docx]

**Supplementary Table 1.** Strains used in this study. All strains carry the *veA1* mutation affecting sporulation. *pabaA1*, *pyroA4*, *riboB2*, *argB2*, *pyrG89, pantoB100, nicA2* and *inoB2* are auxotrophic mutations for p-aminobenzoic acid, pyridoxine, riboflavin, arginine, uracil/uridine, D-pantothenic acid nicotinic acid and inositol respectively. *yA2* and *wA4* are mutations resulting in yellow and white conidiospore colors respectively.

| **Name** | **Genotype** | **Reference** |
| --- | --- | --- |
| TNO2A7 | *TNO2A7: nkuAΔ::argB pyrG89 pyroA4 riboB2* | Nayak *et al*., 2006 |
| PH^OSBP^ | *pyroA4::[pyroA::gpdA^m^_p_::mRFP-PH^OSBP^] inoB2 niiA4 wA4* | Pantazopoulou and Peñalva, 2009 |
| sagA-GFP | *sagA-GFP::AFpyrG nkuAΔ::argB pyroA4 riboB2 pyrG89* | Karachaliou *et al*., 2013 |
| slaB-GFP | *MAD1794:* *slaB-GFP::AFpyrG Δnku::argB pyrG89 pyroA4 argB2* | Araujo-Bazán *et al*., 2008 |
| sagAΔ | *sagAΔ::AFriboB nkuAΔ::argB pyrG89 pyroA4 riboB2* | Karachaliou *et al*., 2013 |
| sagAΔ | *sagAΔ::AFriboB uapAΔ uapCΔ::AFpyrG azgAΔ pabaA1 argB2* | Karachaliou *et al*., 2013 |
| uapA-GFP | *uapA-GFP::AFriboB uapCΔ::AfpyrG nkuAΔ::argB pabaA1 pyroA4 riboB2* | Evangelinos *et al*., 2016 |
| alcA_p_-uapA-GFP | *alcA_p_::uapA-GFP::AFriboB uapCΔ::AfpyrG nkuAΔ::argB pabaA1 pyroA4 riboB2* | Evangelinos *et al*., 2016 |
| prnB-GFP | *prnBΔ::prnB-GFP argB2 pabaA1 yA2* | Tavoularis *et al*., 2001 |
| agtA-GFP | *agtAΔ::agtA-gfp::AFpyrG pyroA4* | Apostolaki *et al*., 2009 |
| fcyB-GFP | *[fcyB-GFP]pBS-argB uapAΔ uapCΔ::AFpyrG azgAΔ argB2 pabaA1* | Vlanti and Diallinas, 2008 |
| furA-GFP | *[gpdA_p_::FurA-GFP]pGEM-panB uapAΔ uapCΔ::AFpyrG azgAΔ fcyBΔ::argB furDΔ::riboB furAΔ::riboB cntAΔ::riboB pantoB100 pabaA1* | Krypotou *et al*., 2015 |
| furE-GFP | *[gpdA_p_::FurE-GFP]pGEM-panB uapAΔ uapCΔ::AFpyrG azgAΔ fcyBΔ::argB furDΔ::riboB furAΔ::riboB cntAΔ::riboB pantoB100 pabaA1* | Krypotou *et al*., 2015 |
| furD-GFP | *[gpdA_p_::FurD-GFP]pGEM-panB uapAΔ uapCΔ::AFpyrG azgAΔ fcyBΔ::argB furDΔ::riboB furAΔ::riboB cntAΔ::riboB pantoB100 pabaA1* | Krypotou *et al*., 2015 |
| mCherry-synA GFP-tpmA | *LO2264: mCherry-synA::AFpyrG yA::AFpyroA GFP-tpmA fwA1 pyrG89 pyroA4 nicA2 nkuAΔ::argB* | Taheri-Talesh *et al*., 2008 |
| abpA-mRFP | *MAD1399: abpA-mRFP::AFpyrG yA2 pabaA1 pyrG89* | Araujo-Bazán *et al*., 2008 |
| dnfAΔ | *ZSS5: dnfAΔ::AFriboB nkuAΔ::argB pyrG89 pyroA4* | Schultzhaus *et al*., 2015 |
| dnfBΔ | *ZSS7: dnfBΔ::AFriboB nkuAΔ::argB pyrG89 pyroA4* | Schultzhaus *et al*., 2015 |
| dnfA-GFP | *ZSS3: dnfA-GFP::AFpyrG nkuAΔ::argB pyrG89 pabaA1 pyroA4* | Schultzhaus *et al*., 2015 |
| dnfB-GFP | *ZSS2: dnfB-GFP::AFpyrG nkuAΔ::argB pyrG89 pabaA1 pyroA4* | Schultzhaus *et al*., 2015 |
| stoAΔ | *SNT116: stoAΔ::AFpyrG argBΔ::trpCΔB or argB2 pyrG89? pabaA1* | Takeshita *et al*., 2012 |
| basA1 | *basA1, pyrG89* | Li *et al*., 2007 |
| thiAp-ap1^σ^ | *thiA_~~p~~_::FLAG-ap1^σ^::AFriboB nkuAΔ::argB pyrG89 pyroA4 riboB2* | This study |
| ap2^σ^Δ | *ap2^σ^Δ::AFriboB nkuAΔ::argB pyrG89 pyroA4 riboB2* | This study |
| ap2^μ^Δ | *ap2^μ^Δ::AFpyrG nkuAΔ::argB pyrG89 pyroA4 riboB2* | This study |
| ap3^σ^Δ | *ap3Δ::AFriboB nkuAΔ::argB pyrG89 pyroA4 riboB2* | This study |
| ap2^σ^-GFP | *ap2σ-(5xGA)GFP::AFpyrG nkuAΔ::argB pyrG89 pyroA4 riboB2* | This study |
| ap2^σ^-mRFP | *ap2σ-(5xGA)mRFP::AFpyrG nkuAΔ::argB pyrG89 riboB2 pyroA4* | This study |
| ap2^σ^Δ ap2^μ^Δ | *ap2^σ^Δ::AFriboB Ap2μΔ::AFpyrG pyroA4 riboB2 pyrG89* | This study |
| Ap2^σ^-mRFP Ap2^μ^Δ | *ap2^σ^-(5xGA)mRFP::AFpyrG ap2^μ^Δ::AFriboB nkuAΔ::argB pyrG89 pyroA4 riboB2* | This study |
| thiA_p_-slaB | *thiA_p_-slaB::AFriboB nkuAΔ::argB pyroA4 riboB2 pyrG89* | This study |
| thiA_p_-slaB | *thiA_p_-slaB::AFpyrG nkuAΔ::argB pyroA4 riboB2 pyrG89* | This study |
| claLΔ | *claL::AFpyrG nkuAΔ::argB pyroA4 riboB2 pyrG89* |  |
| thiA_p_-claL | *thiA_p_-claL::AFpyrG nkuAΔ::argB pyroA4 riboB2 pyrG89* | This study |
| thiA_p_-claL | *thiA_p_-claL::AFriboB nkuAΔ::argB pyroA4 riboB2 pyrG89* | This study |
| thiA_p_-claH uapA-GFP | *uapAΔ::uapA-GFP::AFriboB thiA_p_-claH::AFpyroA nkuAΔ::argB pyroA4 pabaA1 pyrG89* | This study |
| thiA_p_-claH DnfA-GFP | *dnfA-GFP::AFpyrG thiA_p_-claH::AFpyroA nkuAΔ::argB pyroA4 pabaA1 pyrG89* | This study |
| thiA_p_-claH DnfB-GFP | *dnfB-GFP::AFpyrG thiA_p_-claH::AFpyroA nkuAΔ::argB pyroA4 pabaA1 pyrG89* | This study |
| thiA_p_-claH-GFP | *thiA_p_-claH::AFpyroA claH-(5xGA)GFP::AFpyrG nkuAΔ::argB pyrG89 pyroA4 riboB2* | This study |
| thiA_p_-basA | *thiA_p_-basA::AFriboB nkuAΔ::argB pyroA4 riboB2 pyrG89* | This study |
| thiA_p_-basA | *thiA_p_-basA::AFpyrG nkuAΔ::argB pyroA4 riboB2 pyrG89* | This study |
| uapA-GFP thiA_p_-slaB | *uapA-GFP thiA_p_-slaB::AFpyrG nkuAΔ::argB pyroA4 pyrG89* | This study |
| uapA-GFP thiA_p_-claL | *uapA-GFP thiA_p_-claL::AFpyrG nkuAΔ::argB pyroA4 pyrG89* | This study |
| ap2^σ^-GFP mCherry-synA | *ap2^σ^-(5xGA)GFP::AFpyrG mCherry-synA::AFpyrG pyroA4* | This study |
| uapA-GFP ap2^σ^Δ | *uapA-GFP ap2^σ^Δ::AFriboB uapCΔ::AfpyrG nkuAΔ::argB pabaA1* | This study |
| prnB-GFP ap2^σ^Δ | *prnB-GFP ap2^σ^Δ::AfriboB nkuΔ::argB* | This study |
| agtA-GFP ap2^σ^Δ | *agtA-GFP ap2^σ^Δ::AfriboB nkuΔ::argB pabaA1* | This study |
| fcyB-GFP ap2^σ^Δ | *[fcyB-GFP]pBS-argB uapAΔ uapCΔ::AFpyrG azgAΔ ap2^σ^Δ::AFriboB pabaA1 pyroA4* | This study |
| furA-GFP ap2^σ^Δ | *[gpdA_p_::FurA-GFP]pGEM-panB ap2^σ^Δ::AFriboB uapAΔ uapCΔ::AFpyrG azgAΔ fcyBΔ::argB furDΔ::riboB furAΔ::riboB cntAΔ::riboB pantoB100 pabaA1* | This study |
| furE-GFP ap2^σ^Δ | *[gpdA_p_::FurE-GFP]pGEM-panB ap2^σ^Δ::AFriboB uapAΔ uapCΔ::AFpyrG azgAΔ fcyBΔ::argB furDΔ::riboB furAΔ::riboB cntAΔ::riboB pantoB100 pabaA1* | This study |
| furD-GFP ap2^σ^Δ | *[gpdA_p_::FurD-GFP]pGEM-panB ap2^σ^Δ::AFriboB uapAΔ uapCΔ::AFpyrG azgAΔ fcyBΔ::argB furDΔ::riboB furAΔ::riboB cntAΔ::riboB pantoB100 pabaA1* | This study |
| dnfA-GFP ap2^σ^Δ | *dnfA-GFP::AFpyrG ap2^σ^Δ::AFriboB nkuAΔ::argB pyrG89 pyroA4* | This study |
| dnfB-GFP ap2^σ^Δ | *dnfB-GFP::AFpyrG ap2^σ^Δ::AFriboB nkuAΔ::argB pyrG89 pyroA4* | This study |
| dnfAΔ ap2^σ^Δ | *ap2^σ^Δ::AFriboB dnfAΔ::AFriboB pabaA1* | This study |
| dnfBΔ ap2^σ^Δ | *ap2^σ^Δ::AFriboB dnfBΔ::AFriboB pabaA1 pyroA4* | This study |
| ap2^σ^-GFP dnfAΔ | *ap2^σ^-(5xGA)GFP::AFpyG dnfAΔ::AFriboB nkuAΔ::argB pyrG89 pyroA4* | This study |
| ap2^σ^-GFP dnfBΔ | *ap2^σ^-(5xGA)GFP::AFpyrG dnfBΔ::AFriboB nkuAΔ::argB pyrG89 pyroA4* | This study |
| stoAΔ ap2^σ^Δ | *stoAΔ::AFpyrG ap2^σ^Δ::AFriboB pabaA1* | This study |
| sagAΔ ap2^σ^Δ | *sagAΔ::AFriboB ap2^σ^Δ::AFriboB uapAΔ pabaA1 pyroA4* | This study |
| alcA_p_-uapA-GFP ap2^μ^Δ | *alc_p_::uapA-GFP::AFriboB αp2^μ^Δ::AFpyrG riboB2 nkuAΔ::argB pyroA4* | This study |
| ap2^σ^Δ ap3^σ^Δ | *ap2^σ^Δ::AFriboB ap3^σ^Δ::AFriboB uapAΔ uapCΔ::AFpyrG pabaA1* | This study |
| ap2^σ^Δ thiA_p_-ap1^σ^ | *thiAp-ap1^σ^Δ::AFriboB Ap2^σ^Δ::AFriboB uapAΔ pabaA1* | This study |
| slaB-GFP ap2^σ^Δ | *slaB-GFP::AFpyrG ap2^σ^Δ::AFriboB nkuAΔ::argB pyroA4* | This study |
| sagA-GFP ap2^σ^Δ | *sagA-GFP::AFpyrG ap2^σ^Δ::AFriboB pabaA1 pyroA4* | This study |
| abpA-mRFP ap2^σ^Δ | *ap2^σ^Δ::AFriboB abpA-mRFP::AFpyrG pabaA1* | This study |
| mCherry-synA ap2^σ^Δ | *ap2^σ^Δ mCherry-SynA GFP-TmpA yA2* | This study |
| ap2^σ^-mRFP sagA-GFP | *ap2^σ^-(5xGA)mRFP::AFpyrG sagA-GFP::AFpyrG pyroA4 riboB2* | This study |
| ap2^σ^-mRFP slaB-GFP | *ap2^σ^-(5xGA)mRFP::AFpyrG slaB-GFP::AFpyrG nkuAΔ::argB pyrG89 pyroA4 riboB2 pabaA1* | This study |
| ap2^σ^-mRFP dnfA-GFP | *ap2^σ^-(5xGA)mRFP::AFpyrG dnfA-GFP::AFpyrG nkuAΔ::argB pyrG89 pyroA4* | This study |
| ap2^σ^-mRFP dnfB-GFP | *ap2^σ^-(5xGA)mRFP::AFpyrG dnfB-GFP::AFpyrG nkuAΔ::argB pyrG89 pyroA4* | This study |
| ap2^σ^-mRFP claL-GFP | *ap2σ-(5xGA)mRFP::AFpyrG claL-(5xGA)GFP::AFpyrG nkuAΔ::argB pyrG89 pyroA4* | This study |
| claL-mRFP ap2^σ^-GFP | *claL-(5xGA)mRFP::AFpyrG ap2^σ^-(5xGA)GFP::AFpyrG nkuAΔ::argB pyroA4 riboB2 pyrG89* | This study |
| claL-mRFP | *claL-(5xGA)mRFP::AFpyrG nkuAΔ::argB pyroA4 riboB2 pyrG89* | This study |
| claL-mRFP uapA-GFP | *claL-(5xGA)mRFP::AFpyrG uapAΔ::uapA-GFP nkuAΔ::argB pyroA4 pabaA1 pyrG89* | This study |
| ap2^σ^-mRFP alcA_p_-uapA-GFP | *ap2^σ^-(5xGA)mRFP::AFpyrG uapAΔ::alcA_p_-uapA-GFP::AFriboB nkuAΔ::argB pabaA1 pyroA4 riboB2* | This study |
| ap2^σ^-GFP mCherry-synA | *ap2^σ^-(5xGA)GFP::AFpyrG mCherry-synA::AFpyrG pyroA4* | This study |
| ap2^σ^-GFP abpA-mRFP | *ap2^σ^-(5xGA)GFP::AFpyrG abpA-mRFP::AFpyrG pyroA4* | This study |
| ap2^σ^-mRFP slaB-GFP sagAΔ | *ap2^σ^-(5xGA)mRFP::AFpyrG sagAΔ::AFriboB slaB-GFP::AFpyrG pyroA4* | This study |
| ap2^σ^-mRFP sagA-GFP thiA_p_-slaB | *ap2^σ^-(5xGA)mRFP::AFpyrG sagA-GFP::AFpyrG thiAp-slaB::AFriboB nkuAΔ::argB pyrG89 pyroA4 riboB2* | This study |
| ap2^σ^-GFP basA1 | *ap2σ-(5xGA)GFP::AFpyrG basA1 pyrG89 pyroA4* | This study |
| thiA_p_-basA ap2^σ^Δ | *thiA_p_-basA::AFpyrG* ap2^σ^Δ::AFriboB *nkuAΔ::argB* pyrG89 riboB2 pyroA4 | This study |
| thiA_p_-basA ap2^σ^-GFP | *thiA_p_-basA::AFriboB ap2σ-(5xGA)GFP::AFpyrG nkuAΔ::argB* pyrG89 riboB2 pyroA4 | This study |
| ap2^σ^-GFP mRFP-PH^OSBP^ | *ap2^σ^-(5xGA)GFP::AFpyrG pyroA4::[pyroA::gpdA^m^_p_::mRFP-PH^OSBP^]* | This study |
