# Supplementary material for: The AP-2 complex has a specialized clathrin-independent role in apical endocytosis and polar growth in fungi
Source: eLife. 2017 Feb 21;6:e20083. doi: 10.7554/eLife.20083 (PMC5338921; doi:10.7554/eLife.20083)
Supplement: Supplementary file 2. — DOI: http://dx.doi.org/10.7554/eLife.20083.023 [file elife-20083-supp2.docx]

**Supplementary Table 2**. Oligonucleotides used in this study for cloning purposes

| **Oligonucleotides** | | **Sequence** |
| --- | --- | --- |
|  | |  |
| ***ap2^σ^Δ::AFpyrG / ap2^σ^Δ::AFriboB*** | | |
| ap2σ 5’ SphI F | CGCGGCATGCGAAGAATCGCGTTAATGTCTCGGG | |
| ap2σ 5’ SpeI R | CGCGACTAGTCTTGTCTGATGGCCCGCGGTGCTC | |
| ap2σ 3’ SpeI F | CGCGACTAGTTTATTGTTCATGTGATAAGTGAACGG | |
| ap2σ 3’ NdeI R | CGCGCATATGGAGATACCAGGAGTAGCCCC | |
| AFriboB SpeI F | CGCGACTAGTAAGCTTGATATCACAATCAGC | |
| AFriboB SpeI R | CGCGACTAGTCCCGGGCTGCAGGAATTCGATAAG | |
| AFpyrG SpeI F | CGCGACTAGTGCCTCAAACAATGCTCTTCACCCTC | |
| AFpyrG SpeI R | CGCGACTAGTACTGTCTGAGAGGAGGCACTGATGCG | |
| ***ap2^σ^-(5xGA)GFP::AFpyrG / ap2^σ^-(5xGA)mRFP::AFpyrG*** | | |
| ap2^σ^ 5’ KpnI F | CGCGGGTACCGTCGTAATATAACTCGTACAGCAATC | |
| ap2^σ^ 3’ ORF SpeI NS R | CGCGACTAGTCTCCAGTTTATCCAGATGTTCTAGCC | |
| ap2^σ^ 3’ SpeI F | CGCGACTAGTTTATTGTTCATGTGATAAGTGAACGGCTGC | |
| ap2^σ^ 3‘NotI R | CGCGGCGGCCGCGAGATACCAGGAGTAGCCCCGCTG | |
| 5xGA SpeI F | CGCGACTAGTGGAGCTGGTGCAGGCGCTGGAGCCGGTGCC | |
| AFpyrG SpeI R | CGCGACTAGTACTGTCTGAGAGGAGGCACTGATGCG | |
| ***ap2^μ^Δ::AFpyrG / ap2^μ^Δ::AFriboB*** | | |
| ap2^μ^ 5’ KpnI F | CGCGGGTACCCCTTACCCTCATCCGACTCCGAAC | |
| ap2^μ^ 5’ SpeI R | CGCGACTAGTTTTGCGATTAGGCGTGGTGGTAAGG | |
| ap2^μ^ 3’ SpeI F | CGCGACTAGTGGCTGATGCTAACATATATATTTGCAG | |
| ap2^μ^ 3’ NotI R | CGCGGCGGCCGCCCACTCCCACTGATGACAGTCAATG | |
| AFriboB SpeI F | CGCGACTAGTAAGCTTGATATCACAATCAGC | |
| AFriboB SpeI R | CGCGACTAGTCCCGGGCTGCAGGAATTCGATAAG | |
| AFpyrG SpeI F | CGCGACTAGTGCCTCAAACAATGCTCTTCACCCTC | |
| AFpyrG SpeI R | CGCGACTAGTACTGTCTGAGAGGAGGCACTGATGCG | |
| ***Ap3^σ^Δ::AFpyrG*** | | |
| ap3^σ^ 5’ ApaI F | CGCGGGGCCCGAGTCCGCACTCAGCAGCTC | |
| ap3^σ^ 5’ SpeI R | CGCGACTAGTTTCGGATATAGGCTGATGGTG | |
| ap3^σ^ 3’ SpeI F | CGCGACTAGTGGTTGTACAGCTTCTCCGTG | |
| ap3^σ^ 3’ NdeI R | CGCGCATATGGGGAAGGAAAACAACCAGCTC | |
| ***thiA_p_-ap1^σ^::AFriboB*** | | |
| ap1^σ^ 5’ ApaI F | CGCGGGGCCCGGCAGGACTGCTGTTAGGGAGC | |
| ap1σ 5’ SphI R | CGCGGCATGCTTTGCAGATTGTGGGAAAAGTCGTGG | |
| ap1^σ^ ORF SacII F | CGGCCCGCGGATGGCAATTCAGTAAGACTTGAATGC | |
| ap1^σ^ 3’ NotI R | CGCGGCGGCCGCGGGTAAGTGATCCCTGTTGTCTTAGC | |
| AFriboB SphI F | CCCGGGCATGCAAGCTTGATATCACAATCAGC | |
| AFriboB SphI R | CCCGGGCATGCCCCGGGCTGCAGGAATTCGATAAG | |
| thiA_p_ SphI F | CGGGCATGCCGACCTGGCACCTACAGAAGAATCC | |
| thiA_p_ FLAG SacII R | CGCGCCGCGGCTTGTCATCGTCGTCCTTGTAGTCCAT  GTTGACTCAGTTCAATGGTTCGACTATAG | |
| ***claLΔ::AFpyrG*** | | |
| claL 5’ NcoI F | CGCGCCATGGCCCTCGCATAATCGCATCCTCTAC | |
| claL 5’ SpeI R | CGCGACTAGTCCTCCTTGTAATATAGGATATACGACG | |
| claL 3’ SpeI F | CGCGACTAGTCTGCTGATTCCCTAATATTCTGGCC | |
| claL 3’ NotI R | CGCGGCGGCCGCCCAGGTCAAAGCCGAGGTTGAAG | |
| AFpyrG SpeI F | CGCGACTAGTGCCTCAAACAATGCTCTTCACCCTC | |
| AFpyrG SpeI R | CGCGACTAGTACTGTCTGAGAGGAGGCACTGATGCG | |
| ***thiA_p_-claL::AFpyrG / thiA_p_-claL::AFriboB*** | | |
| claL 5’ NcoI F | CGCGCCATGGCCCTCGCATAATCGCATCCTCTAC | |
| claL 5’ SpeI R | CGCGACTAGTCCTCCTTGTAATATAGGATATACGACG | |
| claL ORF SpeI F | CGCGACTAGTATGGCTGACCGCTTCCCGTCGTTG | |
| claL 3’ NotI R | CGCGGCGGCCGCCCAGGTCAAAGCCGAGGTTGAAG | |
| thiA_p_ XbaI F | CGCGTCTAGACGACCTGGCACCTACAGAAGAATCC | |
| thiA_p_ SpeI R | CGCGACTAGTGTTGACTCAGTTCAATGGTTCGAC | |
| AFpyrG SpeI F | CGCGACTAGTGCCTCAAACAATGCTCTTCACCCTC | |
| AFpyrG XbaI R | CGCGTCTAGAACTGTCTGAGAGGAGGCACTGATGCG | |
| AFriboB SpeI F | CGCGACTAGTAAGCTTGATATCACAATCAGC | |
| AFriboB XbaI R | CGCGTCTAGACCCGGGCTGCAGGAATTCGATAAG | |
| ***claL-(5xGA)GFP::AFpyrG*** | | |
| ClaL 5’ NcoI F | CGCGCCATGGCCCTCGCATAATCGCATCCTCTAC | |
| ClaL ORF NS SpeI R | CGCGACTAGTAACCCCGCTAGCGCCAGGCGCTC | |
| ClaL 3’ SpeI F | CGCGACTAGTCTGCTGATTCCCTAATATTCTGGCC | |
| ClaL 3’ NotI R | CGCGGCGGCCGCCCAGGTCAAAGCCGAGGTTGAAG | |
| 5xGA SpeI F | CGCGACTAGTGGAGCTGGTGCAGGCGCTGGAGCCGGTGCC | |
| AFpyrG SpeI R | CGCGACTAGTACTGTCTGAGAGGAGGCACTGATGCG | |
| ***claHΔ::AFpyrG*** | | |
| ClaH 5 ApaI F | CGCGGGGCCCGGCAAGTACCTTGTCCTTCAAATGG | |
| ClaH 5 SpeI R | GATGACTAGTGTTGCAGCTGTGAAGTTG | |
| ClaH ORF2 SpeI F | CGCGACTAGTGAGGAACTGGGTGATATTGTCCGACC | |
| ClaH ORF2 NotI R | CGCGGCGGCCGCGCAGTGGCAACAACCTGGTCAATGAGAG | |
| AFpyrG SpeI F | CGCGACTAGTGCCTCAAACAATGCTCTTCACCCTC | |
| AFpyrG SpeI R | CGCGACTAGTACTGTCTGAGAGGAGGCACTGATGCG | |
| ***thiA_p_-claH::AFpyroA*** | | |
| ClaH 5 ApaI F | CGCGGGGCCCGGCAAGTACCTTGTCCTTCAAATGG | |
| ClaH 5 SpeI R | GATGACTAGTGTTGCAGCTGTGAAGTTG | |
| ClaH ORF SpeI F | CGCGACTAGTATGGCTCCTCTTCCCATCAAATTCAC | |
| ClaH ORF NotI R | CGCGGCGGCCGCCTTGTTGATTGTCTCTGGCGTCCTC | |
| AFpyroA XbaI F | CGCGTCTAGAGGACATCAGATGCTGGATTAC | |
| AFpyroA SpeI R | CGCGACTAGTGCGAGTGTCTACATAATGAAGG | |
| thiA_p_ XbaI F | CGCGTCTAGACGACCTGGCACCTACAGAAGAATCC | |
| thiA_p_ SpeI R | CGCGACTAGTGTTGACTCAGTTCAATGGTTCGAC | |
| ***thiA_p_-basA::AFpyrG / thiA_p_-basA::AFriboB*** | | |
| basA 5’ ApaI F | GCGCGGGCCCGAGCGTACTCTTCAGGTGACCCTTG | |
| basA 5’ SpeI R2 | CGCGACTAGTGCAACGTCAATTAGGACGTCGG | |
| basA ORF SpeI F | CGCGACTAGTATGGCTACAAACACAACTTTGCTCTATGATC | |
| basA 3’ NotI R | CGCGGCGGCCGCCGTGGATCGGTTAGGCATGCATATG | |
| thiA_p_ XbaI F | CGCGTCTAGACGACCTGGCACCTACAGAAGAATCC | |
| thiA_p_ SpeI R | CGCGACTAGTGTTGACTCAGTTCAATGGTTCGAC | |
| AFpyrG SpeI F | CGCGACTAGTGCCTCAAACAATGCTCTTCACCCTC | |
| AFpyrG XbaI R | CGCGTCTAGAACTGTCTGAGAGGAGGCACTGATGCG | |
| AFriboB SpeI F | CGCGACTAGTAAGCTTGATATCACAATCAGC | |
| AFriboB XbaI R | CGCGTCTAGACCCGGGCTGCAGGAATTCGATAAG | |
| ***thiA_p_-slaB::AFpyrG / thiA_p_-slaB::AFriboB*** | | |
| slaB 5 KpnI F2 | CGCGGGTACCCGATGATTGAGATATCCCGCCGGTC | |
| slaB 5 SpeI R3 | CGCGACTAGTCAGACCTCCTAAAGTCCGCGGGTCTTG | |
| slaB ORF SpeI F2 | CGCGACTAGTATGAGTCGGTAGGTAATTGGGGACTG | |
| slaB ORF SacI R | CGCGGAGCTCCATACTTGCTTCTCCATGTGTTGAC | |
| thiA_p_ XbaI F | CGCGTCTAGACGACCTGGCACCTACAGAAGAATCC | |
| thiA_p_ SpeI R | CGCGACTAGTGTTGACTCAGTTCAATGGTTCGAC | |
| AFpyrG SpeI F | CGCGACTAGTGCCTCAAACAATGCTCTTCACCCTC | |
| AFpyrG XbaI R | CGCGTCTAGAACTGTCTGAGAGGAGGCACTGATGCG | |
| AFriboB SpeI F | CGCGACTAGTAAGCTTGATATCACAATCAGC | |
| AFriboB XbaI R | CGCGTCTAGACCCGGGCTGCAGGAATTCGATAAG | |

*ap2^σ^-(5xGA)GFP::AFpyrG, claL-(5xGA)GFP::AFpyrG* and *ap2^σ^-(5xGA)mRFP::AFpyrG* constructs carry a 5x Gly-Ala (5xGA) linker, amplified together with GFP or mRFP and *AFpyrG* from plasmids p1439, or p1491 respectively (Szewczyk *et al*., 2006).
